# Evaluation of Short and Long Term Cold Stress Challenge of Nerve Grow Factor, Brain-Derived Neurotrophic Factor, Osteocalcin and Oxytocin mRNA Expression in BAT, Brain, Bone and Reproductive Tissue of Male Mice Using Real-Time PCR and Linear Correlation Analysis

**DOI:** 10.3389/fphys.2017.01101

**Published:** 2018-01-11

**Authors:** Claudia Camerino, Elena Conte, Roberta Caloiero, Adriano Fonzino, Mariarosaria Carratù, Marcello D. Lograno, Domenico Tricarico

**Affiliations:** ^1^Department of Basic Medical Sciences, Neurosciences and Sense Organs, University of Bari Aldo Moro, Bari, Italy; ^2^Department of Pharmacy–Drug Sciences, University of Bari Aldo Moro, Bari, Italy

**Keywords:** osteocalcin, nerve growth factor, brain-derived neurotrophic factor, oxytocin, gene expression, cold stress

## Abstract

The correlation between the *Ngf/p75ntr-Ntrk1* and *Bdnf*, Osteocalcin-*Ost*/*Gprc6a* and Oxytocin-*Oxt/Oxtr* genes, was challenged investigating their mRNA levels in 3 months-old mice after cold-stress (CS). Uncoupling protein-1 (*Ucp-1)* was used as positive control. Control mice were maintained at room temperature T = 25°C, CS mice were maintained at T = 4°C for 6 h and 5-days (*N* = 15 mice). RT-PCR experiments showed that *Ucp-1* and *Ngf* genes were up-regulated after 6 h CS in brown adipose tissues (BAT), respectively, by 2 and 1.5-folds; *Ucp-1* was upregulated also after 5-days, while *Ngfr (p75ntr)* and *Ntrk1* genes were downregulated after 6 h and 5-days CS in BAT. *NGF* and *P75NTR* were upregulated in bone and testis following 5-days, and *P75NTR* in testis after 6 h CS. *Bdnf* was instead up-regulated in bone following 5-days CS and down-regulated in testis. *OST* was upregulated by 16 and 3-fold in bone and BAT, respectively, following 5-days CS. *Gprc6a* was upregulated after 6 h in brain, while *Bglap* (*Ost)* gene was downregulated. *Oxt* gene was upregulated by 5-fold following 5-days CS in bone. *Oxtr* was upregulated by 0.5 and 0.3-fold, respectively, following 6 h and 5-days CS in brain. *Oxtr* and *Oxt* were downregulated in testis and in BAT. The changes in the expression levels of control genes vs. genes following 6 h and 5-days CS were correlated in all tissues, but not in BAT. Correlation in BAT was improved eliminating *Ngfr (p75ntr)* data. The correlation in brain was lost eliminating *Oxtr* data. In sum, *Ucp-1* potentiation in BAT after cold stress is associated with early *Ngf*-response in the same tissue and trophic action in bone and testis. In contrast, BDNF exerts bone and neuroprotective effects. Similarly to *Ucp-1, Bglap* (*Ost)* signaling is enhanced in bone and BAT while it may exert local neuroprotective effects thought its receptor. *Ngfr (p75ntr)* regulates the adaptation to CS through a feed-back loop in BAT. *Oxtr* regulates the gene-response to CS through a feed-forward loop in brain. Overall these results expand the understanding of the physiology of these molecules under metabolic thermogenesis.

## Introduction

Nerve Growth Factor *(Ngf)*/Brain-derived Neurotrophic Factor (*Bdnf*), osteocalcin (*Bglap*) and oxytocin (*Oxt*) share common effects regulating energy, bone mass, reproduction and neuronal functions, suggesting a coordinated regulation (Fulzele et al., 2010; Oury et al., 2011; Camerino et al., 2012; Karsenty, 2012).

In brief, the carboxylated osteocalcin has local effect leading to bone remodeling stimulating osteoblast, while the uncarboxylated form of osteocalcin acts as a hormone regulating insulin secretion and sensitivity (Ferron et al., 2010; Fulzele et al., 2010; Oury et al., 2011; Karsenty, 2012). The hormonal action of osteocalcin is mediated by the GPRC6A receptor which is expressed in various tissues but it seems absent in brain and ovary. The uncarboxylated osteocalcin acts on the CNS enhancing the synthesis of monoamine neurotransmitters, inhibits GABA synthesis, prevents anxiety and depression, favoring memory independently of its peripheral functions in mice (Ferron et al., 2010; Karsenty, 2011; Oury et al., 2013).

The neurotrophins NGF and BDNF besides their classical role in neurogenesis and in synaptic plasticity (Yano and Chao, 2000), are implicated in energy, reproduction and bone metabolism in mice (Rios et al., 2001; Yamashiro et al., 2001; Yao et al., 2002; Camerino et al., 2012). Specifically, NGF and BDNF levels are significantly altered in metabolic syndrome and also in stress conditions (Unger et al., 2007; Sornelli et al., 2009).

The neuropeptide oxytocin is a well-known hormone triggering both central and peripheral effects and social behavior. Oxytocin is anorexigenic and regulates energy metabolism as suggested by the fact that murine models deficient in oxytocin develop late on set obesity, insulin resistance and low sympathetic tone, and regulate bone formation. Indeed, intraperitoneal injection with Oxytocin negatively modulates adipogenesis while promoting osteogenesis. Oxytocin and its receptor are effective in bone. This latter effect of Oxytocin is exerted peripherally since the neuropeptide cannot cross the Blood Brain Barrier (BBB) (Camerino, 2009a).

BDNF/NGF/Osteocalcin and Oxytocin are also involved in the physiologic adaptation of the body to stressful condition. For example, acute stress like restrain immobilization, in contrast to milder stressors consistently induced highly elevated plasma osteocalcin in rats together with epinephrine, norepinephrine and corticosteroid. Removal of epinephrine by adrenal medullectomy did not alter this response (Patterson-Buckendahl et al., 1995). Moreover, osteocalcin signaling in myofibers is necessary for adaptation to exercise doubling during strength training (Mera et al., 2016). In a model of cold-restrained stressed rats also BDNF increases in brain and this effect is ameliorated by pre-treatment with citalopram (Garabadu et al., 2015), while plasmatic and hypothalamic concentration of oxytocin increases with different kinetics after repeated exposure to an intensive stressor (Danevova et al., 2013).

Brown adipose tissue (BAT), besides its role in not shivering thermogenesis and energy dissipation, is essential for the determination of insulin sensitivity and regulation of energy metabolism and BAT activity has been demonstrated to positively correlate with bone mass. In humans BAT activity correlated negatively with impairment in energy metabolism seen with aging, diabetes and obesity. These conditions are associated with a decrease in bone mass, an increase in fat volume and an increase in fractures. These observations suggest that energy metabolism regulates bone turnover (Rahman et al., 2013). Importantly research has shown a certain plasticity of fat tissue leading to a phenomenon called browning of white adipose tissue depots or skeletal muscle resulting in beige adipocytes. Browning occurs during events such as cold exposure and strength training. Interestingly, rats undergoing cold exposure, when BAT is highly activated, exhibit reductions in circulating concentrations of bone formation markers indicating that cold stress may reduce osteoblast function (Rahman et al., 2013). On this regard, BAT deficient Misty mice shows impaired bone remodeling and decreased bone markers parameters after cold exposure (Motyl et al., 2012). Differently, *in vitro* studies show that short term moderate hypothermia stimulates alkaline phosphatase activity and osteocalcin expression in osteoblasts (Aisha et al., 2014).

The ability of BAT to dissipate energy in the form of heat occurs specifically through the actions of uncoupling protein-1 (UCP-1). The presence of UCP-1 during mild cold stress is protective of bone mass, thus UCP-1 is a critical mediator of BAT's anabolic action on bone. Indeed, mice lacking UCP-1 show a significant reduction in linear and radial grow of bone under chronic mild stress conditions, a change that is absent when UCP-1^−/−^ mice are kept for under thermo-neutral conditions. The action of UCP-1 in response to cold stress on bone appears to be indirect since no protein expression was found in bone by western blot analysis (Nguyen et al., 2015). Thus, the stimulation of UCP-1 activity triggered by cold exposure appears to result in the activation of a protective pathway capable of stimulating osteoblast activity and preserving bone architecture.

Despite the critical role of NGF/BDNF/Osteocalcin and Oxytocin in mediating bone homeostasis, energy and reproductive function data on the effects of prolonged cold exposure are missing in the literature as well as their potential relationship with UCP-1 function.

In a previous work, the transcript levels of *Ngf, Bdnf*, Bglap (*Ost)*, and *Oxt* genes as well as of their receptors Ngfr *(p75ntr)/Ntrk1, Ntrk2, Gprc6a*, and *Oxtr* in brain, bone, white/brown adipose tissue (WAT/BAT) and reproductive organs of 3 months old female and male wt mice were evaluated by RT-PCR experiments (Camerino et al., 2016).

Since Osteocalcin and Oxytocin favor physiological functions that decline with age, as male fertility, memory, muscle maintenance and adaptation to exercise (Elabd et al., 2014; Mera et al., 2016), and NGF/BDNF are involved in stress-related conditions, in order to gain better insight in the physiology of NGF/BDNF, osteocalcin and oxytocin and to explore the possibility that BAT and UCP-1 anabolic function on bone may be mediated by NGF/BDNF, osteocalcin and oxytocin we challenged this previous system investigating *Ngf* and its receptors Ngfr *(p75ntr)* and *Ntrk1, Bdnf*, Bglap (*Ost)* and its receptor *Gprc6a, Oxt* ant its receptor *Oxtr* mRNA, from 3 months-old mice in bone, brain, BAT and testis after 6 h and 5 days of cold stress (CS). *Ucp-1* gene in BAT was used as a positive control. These studies could provide insight about the role of a specific gene in regulating the response of a specific tissue to thermogenic stress and to develop a therapeutic strategy to counterbalance states of increased energy demand following patho-physiological conditions.

## Materials and methods

### Animals care and cold exposure

The protocol design was based on the Replacement, Reduction and Refinement principles described in the Italian D.L. 4 march 2014, n. 26, and 2010/63/EU law on Animal Protection Used for Scientific Experiments. The experimental protocol was submitted during 2012 and approved by the competent Authority of the University of Bari and Italian Health Department of Rome with silent consent. Male 3 months-old C57BL/6N mice were used. They were singly housed in conventional cages with a 12:12-h light/dark cycle with free access to water and standard diet. After 1 week of adaption, they were randomly divided into three groups: room temperature (RT = 25°C) (*N* of mice = 5), cold exposure (CS) (*T* = 4°C) for 6 h (*N* of mice = 5) and 5 days (*N* of mice = 5). Diet and light conditions were identical among the three groups throughout the study. Body weight and food intake in both RT and cold groups were measured daily. Fat pad was measured after surgical removal of the abdominal fat. The animals were sacrificed by an overdose of Zoletil 100 (200 mg/kg body weight) followed by cervical dislocation. The blood was collected from the heart. Subsequently, from each animal the following organs where quickly isolated and weighed: whole brain, abdominal white adipose tissue (WAT), interscapular BAT, sexual organs (testicles) and femora. All organs were frozen in liquid nitrogen and stored at −80°C for RNA extraction. Femora were dissected from 3-month-old mice and prepared as previously described (Lecka-Czernik, 2012; Camerino et al., 2016).

### Real-time PCR experiment

The RNA extraction and pre-amplification protocols were performed as previously described (Tricarico et al., 2002, 2003, 2013; Cetrone et al., 2014; Mele et al., 2014; Camerino et al., 2016). The mRNA expression of the genes was normalized to the best housekeeping genes *Eef2* selected within β*-Actin, Hprt1*, 2 beta-microglobulin*, Gapdh*, and β*-Actin* by BestKeeper version 1. TaqMan Hydrolysis primer and probe gene expression were designed and synthesized from Thermo fisher scientific and it are described in Table 1. With the exception for β*-Actin* (primer For: 5′-CCAGATCATGTTTGAGACCTTCAA-3, primer Rev: 5′-CATACAGGGACAGCACAGCCT-3, probe: VIC-ACC CCA GCC ATG TAC GTA-MGB, the sequence target was NM_007393.5, with a amplicon length of 71 pb and a assay location in position 469). The RT-PCR experiments were performed in agreement with the MIQE guidelines for qPCR (Bustin et al., 2009).

**Table 1:** Quantitative real-time PCR assay.

**Gene name**	**Gene symbol**	**Assay ID of Therm. Fisher. Scient**.	**RefSeq amplificated**	**Assay location**	**Amplicon Length**
Uncoupling protein 1 (mitochondrial, proton carrier)	*Ucp-1*	Mm01244861_m1	NM_009463.3	1049	73
Nerve growth factor	*Ngf*	Mm01192897_m1	NM_001112698.2	163	79
			NM_013609.3	290	79
Nerve growth factor receptor (TNFRsuperfamily, member 16)	*Ngfr*	Mm01309638_m1	NM_033217.3	1129	64
Neurotrophic tyrosine kinase, receptor, type 1	*Ntrk1*	Mm01219406_m1	NM_001033124.1	740	65
G protein-coupled receptor, family C, group 6, member A	*Gprc6a*	Mm01192897_m1	NM_153071.1	221	78
Bone gamma-carboxyglutamate protein 3	*Bglap3*	Mm01741771_g1	NM_001305448.1		77
			NM_001305449.1		77
			NM_031368.5		77
			NM_001305450.1		77
BRAIN derived neurotrophic factor	*Bdnf*	Mm 04230607_s1	NM_001048139.1	3692	92
			NM_001048141.1	3532	92
			NM_001048142.1	3540	92
			NM_001285416.1	3609	92
			NM_001285417.1	3406	92
			NM_001285418.1	3397	92
			NM_001285419.1	3386	92
			NM_001285420.1	3274	92
			NM_001285421.1	3472	92
			NM_001285422.1	3654	92
			NM_007540.4	3833	92
			NM_001033124.1	740	65
Oxytocin	*Oxt*	Mm00726655_s1	NM_011025.4	424	63
Oxytocin receptor	*Oxtr*	Mm01182684_m1	NM_001081147.1	934	93
Eukaryotic translation elongation factor 2	*Eef2*	Mm 01171434_g1	NM_007907.2	103	74
Hypoxanthine guanine phosphoribosyl transferase	*Hprt1*	Mm00446968_m1	NM_013556.2	630	65
Beta-2 microglobulin	*B2m*	Mm 00437762_m1	NM_009735.3	111	77
Glyceraldehyde-3-phosphate dehydrogenase	*Gapdh*	Mm 99999915_g1	NM_001289726.1	117	107
			NM_008084.3	265	107

### Statistics

Significance between mean were evaluated by *student t-*test (*p* < 0.05). Significance within and between data groups was evaluated by ONE WAY ANOVA and the Bonferroni's test for the evaluation of the effects a specific data group on variance (*p* < 0.05). Regression analysis was used to find equations that fit the change of the gene expression data in control mice vs. mice following cold stress challenge. The linear regression equation y = mx + b was used and the Pearson's Correlation Coefficient was also calculated using Excell Software (Microsoft) electronic datasheet. The regression analysis was performed in the presence of all gene expression data in all tissues. To evaluate the contribution of a specific gene to the observed correlation between variables, the equations and the Correlation Coefficient (*R*^2^) were computed in the absence of the expression data for each gene.

## Results

### Effects of short-term (6 h) and long-term (5 days) cold exposure on the mRNA levels of *NGF/P75NTR/NTRK1, OST/GPRC6A, BDNF, OXY/OXTR* and *UCP1* genes in BAT, bone, brain and testis

**Figure 1:**
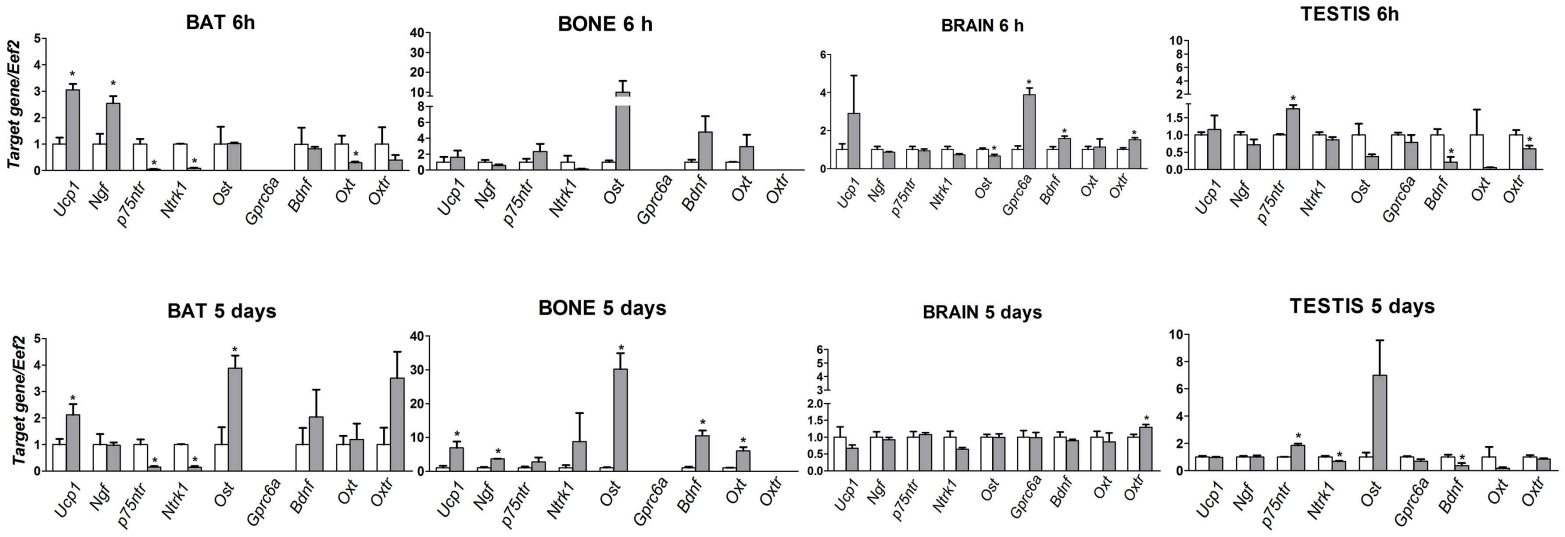
mRNA levels of *Ucp-1, Ngf, Ngfr (p75ntr), Ntrk1, Bdnf, Oxt, Oxtr, Bglap(Ost)* and *Gprc6a* genes in bone, brain, BAT and testis in mice following 6 h and 5 days of cold stress. The data were expressed as mean ± SEM of a minimum of 4 and a maximum of 7 samples for each bar. ^*^Data significantly different within groups by ONE WAY ANOVA and Bonferroni test (*p* < 0.05).

*Ucp-1* and *Ngf* mRNA levels were significantly enhanced after 6 h cold stress in BAT respectively by 2 (*p* = 0.001) and 1.5-fold (*p* = 0.013) vs. controls, while *Ngfr (p75ntr)* and *Ntrk1* genes were down-regulated by 0.95 and 0.92 folds, respectively (*p* = 0.001 and *p* = 0.0002) (Figure 1). The potentiation of the *Ucp-1* gene was maintained in BAT after 5-days of cold stress (*p* = 0.048), while the *Ngf* gene was not affected and mRNAs of its receptors *Ngfr (p75ntr)* and *Ntrk1* were down-regulated by 0.85 and 0.84 folds, respectively (*p* = 0.002 and *p* = 0.0001) vs. controls (Figure 1). The *Ngfr (p75ntr)/Ntrk1* receptor genes were not affected in bone and brain, while the mRNA level of the *Ngf* gene was significantly enhanced in bone 2.6-fold and unchanged in brain following 5 days CS (Figure 1). *Ngfr (p75ntr)* significantly increases in testis after 6 h and 5days of CS by 0.76 (*p* = 0.0005) and 0.83-fold (*p* = 0.0017) vs. controls while *Ngf* and *Ntrk1*were unaffected (Figure 1).

*Bdnf* gene expression shows an opposite trend. The mRNA levels of *Bdnf* enhanced significantly by 9.5-fold (*p* = 0.013) in bone following 5-days of cold stress vs. controls but was not affected in BAT (Figure 1). A mild significant enhancement of the *Bdnf* mRNA level was observed in brain (*p* = 0.0263) after 6 h (Figure 1). The mRNA levels of the *Bdnf* gene significantly decreased by 0.78 (*p* = 0.0142) and 0.6 (*p* = 0.048) folds, respectively, after 6 h and 5-days of CS in testis.

Osteocalcin mRNA levels increased significantly in bone by 16-fold (*p* = 0.016) following 5-days of cold stress vs. controls (Figure 1). Osteocalcin is also upregulated in BAT by 3-fold (*p* = 0.01) after 5-days. Osteocalcin receptor gene is upregulated by 3-fold (*p* = 0.0294) after 6 h in brain, while the osteocalcin gene is downregulated by 0.4-fold; these genes were not affected after 5-days of cold stress (Figure 1). *Ost/Gprc6a* mRNA levels were not significantly affected in testis following CS.

*Oxt* mRNA enhanced by 5 (*p* = 0.416) folds, following 5 days CS vs. controls in bone. The *Oxtr* mRNA levels enhanced significantly by 0.5 (*p* = 0.0058) and 0.3 (*p* = 0.0045) folds, respectively, following 6 h and 5 days CS vs. controls in Brain. *Oxtr* gene was instead down-regulated after 6 h of CS in testis by 0.4-folds (*p* = 0.043) vs. controls, while *Oxt* mRNA levels decrease in BAT by 0.7 (*p* = 0.0296) vs. controls after 6 h of CS.

Regression analysis showed that changes in the expression levels of the genes of control mice vs. mice following 6 h and 5 days of cold stress were linearly correlated in brain, bone and testis showing positive slope and a coefficient of correlation close to 1 in all tissues (Table 2). In contrast, the linear correlation is lost following 6 h and 5 days of cold stress in BAT. In this tissue, the calculated coefficients of correlation were lower than 0.2 (Table 2). In order to understand the relative contribution of a specific gene to this effect in BAT, we performed correlation analysis between the two variables computing the linear regression equation eliminating the expression data for each single gene in Excell electronic datasheet. No differences were found in BAT on correlation parameters computing the equations in the absence of *Bdnf, Oxt, Bglap (Ost)* genes and their receptors, and of the *Ngf* gene. In contrast, upon the elimination of Ngfr (*p75ntr)* expression data from the correlation electronic datasheet significantly improved the *R*^2^ of the equation in BAT (Table 2).

**Table 2:** Linear regression table of the changes of gene expression of controls mice vs. mice following cold stress.

**Tissues**	**6 h of cold stress**	**5 days of cold stress**
BONE all genes data	y = 0.2295x + 0.241 *R*^2^ = 0.9387	y = 0.1045x + 0.2404 *R*^2^ = 0.9183
BRAIN all genes data BRAIN in the absence of *Oxtr* gene data	y = 0.8531x – 0.0423 *R*^2^ = 0.9338 y = 0.859x + 0.2365 *R*^2^ = 0.3643	y = 1.1539x – 0.0935 *R*^2^ = 0.9839 y = 0.9816x − 0.026 *R*^2^ = 0.9687
TESTIS all genes data	y = 0.535x + 0.0998 *R*^2^ = 0.913	y = 0.552x + 0.1281 *R*^2^ = 0.9083
BAT all genes data BAT in the absence of *Ngfr(p75ntr)* gene data	y = 10.124x + 7.048 *R*^2^ = 0.038 y = 93.479x − 3.982 *R*^2^ = 0.7028	y = 0.2858x + 0.1651 *R*^2^ = 0.123 y = 1.702x − 0.023 *R*^2^ = 0.917

Computing the linear regression equation in brain upon elimination of the *Oxtr* data from the correlation electronic datasheet led to the loss of correlation between the expression levels of the genes of controls mice vs. mice following 6 h cold stress (Table 2), supporting the key contribution of this gene in this animal model of CS. No changes were observed following extraction of other genes data from the correlation electronic datasheet in all other tissues.

**Figure 2:**
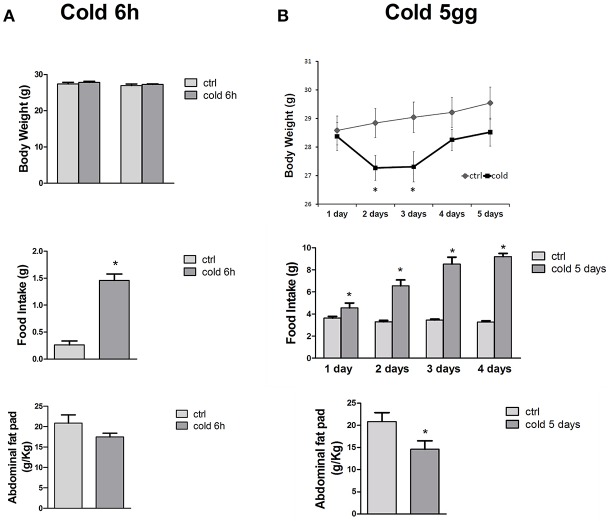
**(A,B)** Body-weight changes, food intake and fat pad content in mice following 6 h and 5 days of cold stress. The cold stress challenge of 6 h induced a significant increase of the food intake in mice vs. control mice. The long term cold stress of 5 days, significantly reduced the abdominal fat pad content and enhanced the food intake of the mice vs. control mice. The data were expressed as mean ± SEM of a minimum of 4 and a maximum of 7 mice. ^*^Data significantly different vs. controls by student test (*p* < 0.05).

The cold stress challenge for 6 h induced a significant 5-fold increase of the food intake in mice vs. control mice, as expected, with no significant changes in the abdominal fat pad content and body weight of the animals (Figure 2A). Exposure to 5 days of cold, significantly reduced the abdominal fat pad content by 1.33-fold and enhanced the food intake by 3-fold of the CS treated mice vs. control mice without significantly affecting the mice body-weight (Figure 2B).

## Discussion

In this study, we show that the expression of *Ngf/Bdnf*, osteocalcin and oxytocin mRNA after cold stress is characterized by early response of these genes in brain and BAT and late response in bone consistent with a fast adaptation to challenges. Differently, in reproductive tissue, we detected a response at both time points.

During cold stress in mice *Ucp-1* increase in BAT is associated with early *Ngf* gene response in the same tissue and down-regulation of *Ngfr (p75ntr)* and *Ntrk1* receptors. *Ucp-1* and *Ngf* in bone are also upregulated but after 5 days of CS. Moreover, a long-term potentiation of NGF receptor gene was found in testis. The upregulation of *Ngf* in BAT suggests that the role of NGF signaling as a regulator of energy reported in untreated mice (Camerino et al., 2016), is observed also after cold stress together with the physiologic role of NGF in fertility (Ratto et al., 2012). It should be of note that in RT-PCR experiments *Ucp-1* gene mRNA was amplified in bone of control mice and following CS, while previous work failed to detect UCP-1 protein by western blot analysis in this tissue (Nguyen et al., 2015). This finding suggests that *Ucp-1* gene is constitutively expressed in bone but the level of the relative functional protein product is finely tuned in bone.

Interestingly, the main role of NGF in reproductive function is further confirmed by linear correlation analysis calculated between the changes of the gene expression in control mice vs. CS mice in testis suggesting that NGF is part of a coordinated response to CS challenge in this tissue. Instead, the lack of correlation between the gene expression in control mice vs. CS mice observed in BAT was found to be dependent on NGF receptor *Ngfr (p75ntr)*, as demonstrated by the fact that computing the linear regression equations upon elimination of the *Ngfr (p75ntr)* expression data significantly improved the *R*^2^ factor (0.7028 at 6 h CS and 0.917 at 5 days CS) of the linear regression equation. These findings suggest that NGF regulates energy in BAT exerting a negative feedback control of energy demand through down-regulation of *Ngfr (p75ntr)* in this tissue following CS. An experimental study shows that plasma NGF levels are increased in patients with metabolic syndrome and obesity (Hristova, 2013) validating our finding of NGF regulating energy metabolism.

Differently, the *Ucp-1* gene potentiation following cold stress is associated with the enhancement of *Bdnf*, signaling in brain after 6 h and bone after 5 days, and down-regulation in testis. These findings are consistent with the role of BDNF signaling as a regulator of bone mass reported in previous studies (Camerino, 2012; Camerino et al., 2012).

Similar to *Ucp-1*, also osteocalcin signaling is potentiated in BAT and in bone following 5 days cold stress to exert local protection to bone against energetic challenges. This finding supports the protective effects of UCP1 on bone. The up-regulation of the osteocalcin receptor gene observed after 6 h of cold stress in brain can be explained by the hormonal proposed action of bone-released osteocalcin as a protector of brain function (Karsenty and Oury, 2014; Camerino et al., 2016).

The evidence that the oxytocin *Oxtr* gene is upregulated in brain consistently at 6 h and 5 days of cold stress suggest that oxytocin plays a main role in this tissue in response to cold stress challenge. OXT emerges as an essential factor regulating the coordinated gene response to the paradigm of cold stress possibly acting as master gene. This is supported by the fact that computing the linear regression equation in the absence of the *Oxtr* expression gene data in Brain (from the electronic data sheets) we report a loss of correlation between the *Ngf/Bdnf, Bglap (Ost)* gene expression of control mice versus gene expression of cold stresses mice.

Oxytocin gene significantly decrease in BAT after 6 h CS but increase in bone after 5 days, supporting the concept of a coordinated axis mediated by oxytocin between energy and bone (Camerino, 2009a,b; Mosialou et al., 2017).

*Oxtr* and *Ngfr (p75ntr)* genes are up-regulated and down-regulated in brain and BAT respectively also after long term, 5 days cold exposure. With the exception of osteocalcin in BAT, the rest of the genes investigated in this study are unaffected at this time point. This observation highlight that *Oxtr, Ngfr (p75ntr)* and Osteocalcin are adaptive and important in restoring the homeostasis of the body.

The observed enhancement of the food-intake following long term CS compensates for the loss of the abdominal fat pad content thereby re-establishing the normal body of the animals in this condition.

The enhancement of the food intake following CS can be related to the OXT signaling in brain as this factor regulate food consumption. BDNF also regulates food consumption, as conditional KO mice in which BDNF has been centrally deleted show obesity and hyperactivity (Rios et al., 2001). *Bdnf* increase in brain after 6 h of CS, it is neuroprotective and may contribute to the re-establishment of normal body weight in these mice.

The negative feed-back loop of the *Ngfr (p75ntr)* gene expression observed in BAT and the lack of the neurotrophic effects in the presence of a significant *Ucp-1* upregulation following cold stress indicate that the downregulation of the NGF receptor *Ngfr (p75ntr)*, is necessary condition to stimulate a higher food consumption in these mice. NGF stimulates the production of the anorexigenic hormone leptin (Hristova, 2013), and the downregulation of *Ngfr (p75ntr)* probably decrease leptin secretion increasing food intake in this experimental model. While, the upregulation of the *Ngf* gene expression in bone and *Ngfr (p75ntr)* gene expression in testis support the protective role of the NGF signaling in these tissues in this condition.

In sum, the role of NGF signaling as a regulator of energy and reproductive function reported in untreated mice (Camerino et al., 2016), is therefore confirmed following thermogenic stress. In this work, we show that *Ngfr (p75ntr)* regulates the adaptation to cold stress through feed-back loop in BAT. Differently the *Oxtr* regulates the coordinated gene response to cold stress through a feed-forward loop in brain. *Bdnf* activity may exert bone and neuroprotective effects. Osteocalcin exerts local protective effects on bone and neuroprotective effects thought its receptor. The biological advantage of this mechanism is that the lack of effects of a specific gene following pathophysiological conditions, gene downregulation or adaptation to external challenges can be counterbalanced by the action of other genes (Karsenty and Oury, 2012).

The potent protective effects of Osteocalcin and Oxytocin against the thermogenic insult in the tissues considered in this study, are thus promising for developing an effective and safe therapeutic strategy to address states of physiological challenge as nutritional or age-associated detrimental conditions.

## Author contributions

CC elaborated the hypothesis and theory; EC, RC, and AF conducted the experiments; CC, EC, MC and ML analyzed the results; CC and DT designed the studies, wrote the manuscript and all authors approved the manuscript.

### Conflict of interest statement

The authors declare that the research was conducted in the absence of any commercial or financial relationships that could be construed as a potential conflict of interest.
